# Retromuscular, periprosthetic drainage after hernioplasty with sublay mesh reinforcement in ventral hernias results in less retromuscular fluid collections but longer hospital stay and analgetic use with unclear effect on clinical outcome - a randomized controlled trial

**DOI:** 10.1007/s00423-024-03522-6

**Published:** 2024-11-05

**Authors:** Julius Pochhammer, Caroline Ibald, Marie-Pascale Weller, Michael Schäffer

**Affiliations:** 1 Department of Visceral, General, and Thoracic Surgery, Marienhospital Stuttgart, Stuttgart, Germany; 2 Department of Visceral and Thoracic Surgery, University Hospital Schleswig-Holstein, Christian-Albrechts-University Kiel, Kiel Campus, Arnold-Hellerstr. 3, 24105 Kiel, Germany; 3 Department for Thoracic Surgery, Kantonspital St. Gallen, St. Gallen, Switzerland

**Keywords:** Sublay hernia repair, Retromuscular fluid collection seroma, Drainage, SSI, SSO

## Abstract

**Purpose:**

To determine whether periprosthetic drain insertion for hernioplasty using sublay mesh augmentation influences retromuscular fluid collections (RFC) and the clinical course.

**Methods:**

Forty-two patients with open repair of midline hernias (M2-4, W1, European Hernia Society classification) were allocated to groups with or without retromuscular drains. Subcutaneous drainages were used in both groups to avoid confounding from surgical site occurrences due to superficial, subcutaneous fluid collections. The participants underwent clinical and ultrasound assessments on postoperative days (POD) 14 and 30 to detect RFC, subcutaneous seromas, and wound complications. The sample size was estimated using the RFC of a test cohort with drainage; the assumed relevant volume (5 ml) was calculated comprising 84% (mean + 1 SD) of these patients.

**Results:**

In the retromuscular drainage group, the RFC median volume was reduced by 75.2% on POD 14, and by POD 30, no RFC were found [0.2 vs. 25.8 (*p* < 0.001) and 0 vs. 4.0 (*p* = 0.02) on PODs 14 and 30, respectively]. The number of patients with RFC ≥ 5 mL was also significantly lower in the drainage group [4 vs. 12 (*p* = 0.02) and 1 vs. 8 (*p* = 0.02) on PODs 14 and 30, respectively]. No surgical site infections occurred in either group, but retromuscular hematoseroma led to one revision surgery and one needle aspiration in the group without drainage. In the drainage group, a significantly longer hospital stay (6.5 days vs. 4 days; *p* = 0.01) and longer regular analgetic intake (6 vs. 3 days; *p* = 0.03) were observed. Multivariable regression revealed that retromuscular drainage usage was the only independent predictor of the RFC volume.

**Conclusion:**

We found that the use of retromuscular drains after hernioplasty with sublay hernia repair reduced periprosthetic fluid collections in our population but prolonged hospital stay. Whether the reduction of RFC can prevent SSO or revision surgery cannot be determined from our data, the relevance is therefore not assessable. Hence, further larger studies are required to determine the clinical relevance of drains.

## Introduction

Primary or incisional ventral hernias are among the most common indications for procedures in general surgery. Because of the latency in their occurrence, incisional hernias after laparotomy are difficult to determine; however, it is realistically assumed that their occurrence ranges between 9% and 20% [1, 2]. There are several methods of ventral hernia repair, including suture repair, as well as open, laparoscopic, and robotic mesh-reinforced procedures. Of the various approaches for mesh-reinforced procedures according to a Cochrane analysis, none has advantages over the other, but there is a trend toward the retromuscular procedures (modified Rives-Stoppa method) [3, 4].

According to register-based data drainage systems are used in up to 72% of procedures to enable the removal of fluid with the idea to prevent surgical site occurrences (SSOs) [5]. Furthermore, it has been assumed that existing hematomas and consecutive surgical site infections (SSI) can lead to hernia recurrences [6, 7]. Other studies point out the possibility of increased SSI due to bacterial translocation via drainages [8–10]. Complete bacterial biofilm formation has been detected on drainages soon after insertion [11]. In another study, abandonment of drainage did not increase seroma or recurrence [12]. Especially with the sublay mesh position, rapid integration into the well-perfused muscle layer and rapid absorption of liquid are expected. Krpata et al. demonstrated in a retrospective register study that the use of drains did not increase the number of SSIs; however, it decreased the incidence of noninfectious SSOs [13]. Studies have demonstrated that drains extend the length of hospital stay (LOS) and may be associated with increased pain [9, 14, 15]. A Cochrane review has addressed drain placement following hernioplasty including various repairs, however theycould not identify randomized trials and called for further randomized studies for the various aspects of drain insertion [16]. Two recent meta-analyses identified only four randomized studies, most of which did not differentiate between the drainage localizations [17, 18]. The conclusion was that the use of drains does not lead to increased complications.

The present study aimed to determine whether waiving the use of retromuscular drains for ventral hernioplasty of low complexity with sublay mesh reinforcement results in increased fluid collections in the retromuscular space. Fluid collections (seromas) in the subcutaneous, superficial space are common, but separated from the mesh by the reconstructed linea alba. They typically regress without further treatment or after needle aspiration but can cause SSO [19–21]. Therefore, in our study, we drained the superficial space to avoid confounding and focused on the retromuscular layer.

## Materials and methods

This was a two-armed, randomized, controlled clinical trial. Inclusion criteria were abdominal wall hernias of the midline not exceeding 4 cm in width (Small or Medium, Midline for primary hernias; M2-4, W1 for incisional hernias according to the European Hernia Society classification [22]), without previous hernia surgery or defects in the rectus sheath. The maximum vertical length of the hernia was not specified. All underwent electively planned hernioplasty with mesh reinforcement using the retrorectus technique, were age ≥ 18 years, and ableto provide consent to participate in the study. The exclusion criteria were hemostasis disorders due to pathological synthesis disorders and the necessity for lateral component separation [23]. Refusal of the patient’s consent or missing information were also exclusion criteria.

### Perioperative care

Conventional retrorectus hernia repair was performed by six board-certified surgeons using standard procedures between 6/2016 and 1/2018. All patients received preoperative antibiotic prophylaxis, including intravenous cefazolin (2 g) or clindamycin (600 mg) for patients with an allergy. Administration of antibiotic prophylaxis before skin incision was verified using a checklist and was not continued postoperatively. The skin was prepared using an alcohol-based disinfectant in a standard manner (Softasept^®^ N; B. Braun AG, Melsungen, Germany). Low-molecular-weight heparin (LMWH) was administered pre- and postoperatively as thromboembolic prophylaxis. Patients with indications for oral anticoagulation received a weight-adjusted therapeutic dose. Antiplatelet anticoagulation with one drug was continued, and patients with dual antiplatelet therapy were excluded. Before implantation of the mesh, gloves were changed, and the wound was rinsed with polyhexanide (Lavanid^®^ 2; Serag-Wiessner GmbH & Co. KG, Naila, Germany). An Adhesix^®^ mesh (C.R. Bard GmbH, Karlsruhe, Germany) was used for all patients. This type of mesh is made of polypropylene and is lightweight, self-adhering, and non-absorbable. Its self-adhesiveness is provided by a gel coating of polyvinylpyrrolidone and polyethylene glycol, and it can be used without further fixation. Both fascial sheets of the rectus sheath were closed using non-absorbable sutures of polypropylene of size 0 in running, small-bite technique (Ethicon). An abdominal binder was applied immediately in the operating room and recommended to be worn for 2 weeks.

### Treatment arms

In the intervention group, no retromuscular, periprosthetic drainages were inserted, while in the control group, at least one Redon drainage (B. Braun AG) with a diameter of at least 12 charriére was inserted in the retromuscular, periprosthetic space. In the separated subcutaneous space, Redon drainages were applied in both groups. All drains were removed during the postoperative course after the daily output decreased to < 20 mL per 24 h.

### Randomization and blinding

Randomization of the study numbers was performed according to a simple computer-based model before the beginning of the study. Stratification was not performed. Marked cards were kept in opaque envelopes with their corresponding study numbers. The study numbers were assigned depending on the time of inclusion. The investigator who performed ultrasonography examinations was blinded to the allocation.

### Ethical considerations, motivation, and funding

This was an investigator-initiated trial. The study protocol was approved by the Ethics Committee of the Medical Association of Baden-Württemberg in Germany (F-2015-049). The study is registered with the German Clinical Trials Register (DRKS00008856). We conducted the trial using our own resources.

### Main outcome measures

The primary endpoints were sonographically measured volumes of fluid collections, hematoma or seroma in the retromuscular space, behind the linea alba and anterior rectus sheath (retromuscular fluid collection, RFC). The measurement was performed on postoperative days (PODs) 14 and 30 using a GE Healthcare LOGIQ S8 ultrasound system (GE Ultraschall Deutschland GmbH, Solingen, Germany) with an 8-MHz linear probe. Examinations were performed by one trained investigator (J.P.) in a standardized manner and documented with pictures. Subcutaneous fluid collections were detected in the same manner.

### Secondary endpoints

Secondary endpoints were complications within 30 days. These included mesh-associated complications, such as SSIs, according to the Centers for Disease Control and Prevention classification [24], and SSOs resulting in secondary wound healing due to seromas or hematomas. Reoperation or invasive measures and general complications were classified according to the Dindo-Clavien classification [25]. In addition, removal of the last drain, length of inpatient stay, unplanned hospital readmission, postoperative pain, and the necessity for opioid analgesics were assessed.

### Sample size estimation

To perform a sample size estimation, we examined 11 consecutive patients 14 days after hernioplasty with sublay mesh augmentation and the use of drainage in the retromuscular space, following the above mentioned inclusion criteria through ultrasound examination. The detectable RFC of this population had a mean volume of 2.1 mL (range, 0 − 10 mL; standard deviation, ± 2.7 mL). We found no data examining a relationship between postoperative fluid volume and the occurrence of SSO. Therefore, we assumed that a RFC below 5 mL (one standard deviation added to the mean value) would be clinically irrelevant as this volume is found in 84% of patients with drainage. For a first-order error of 5%, at least 19 patients per arm were required to achieve a power of 90% [26, 27].

### Biometric evaluation

All study parameters were recorded on a daily basis and finally analyzed using the JMP 13.1 software (SAS Institute Inc., Cary, NC). The primary analysis was based on the intention-to-treat (ITT) principle to mirror clinical practice. Additionally, an as-treated (AT) analysis was performed for the primary endpoint. Data are displayed in their original scale of measurement. Differences in qualitative data were analyzed using the chi-square or Fischer’s exact test, and quantitative data were analyzed using the Wilcoxon-Mann-Whitney U test or paired t-test with normal distribution. A multivariable analysis consisting of multiple regression analysis with backward elimination was performed to determine the size of the fluid collection on POD 14 with all factors shown in Tables 1 and 2. The procedure was performed repeatedly until no explanatory variable was left that could be removed without markedly worsening the prediction of the endpoint.

**Table 1:** Baseline patient characteristics

	Randomized to retromuscular drainage		
	Yes	No	p-value
Total	21 (50)	21 (50)	
Gender female	12 (57.1)	8 (38.1)	0.21
Age (y)	60.2 ± 12.,3	55.4 ± 14.2	0.24
BMI	29.8 ± 7.0	30.5 ± 5.1	0.54
Comorbidities			
Diabetes mellitus	5 (23.8)	3 (14.3)	0.43
Cardiac (incl. Hypertension)	10 (47.6)	7 (33.3)	0.35
Renal	0	2 (9.5)	0.15
Pulmonary	4 (19.1)	5 (23.8)	0.71
ASA ≥ 3	7 (33.3)	4 (19.5)	0.53
Smoking habits	6 (28.6)	2 (9.5)	0.12
Therapeutic dosage of LMWH	5 (23.8)	7 (33.3)	0.73
Ongoing PAI	6 (28.6)	3 (14.3)	0,45

Data are reported as absolute numbers (percentage) or mean ± standard deviation; p-values are calculated using the t-test for continuous data and Pearson´s chi-squared test for categorical data

*BMI*, body mass index; *LMWH*, low molecular weight heparins

**Table 2:** Perioperative characteristics

	Randomized to retromuscular drainage		
	Yes	No	p-value
Total	21 (50)	21 (50)	
Size of mesh (cm^2^)	180 (98–450)	180 (100–500)	0.11
Length of skin incision (cm)	12 (8–21)	11 (6–18)	0.07
Duration of operation (min)	85.0 ± 27.6	98.6 ± 34.3	0.21
Adhesiolysis (compared to length of operation)			
None	6 (28.6)	11 (52.4)	0.03
− 2%	15 (71.4)	7 (33.3)	
2 − 5%	0	3 (14.3)	
> 5%	0	0	
Suture of bowel serosa	0	1 (4.8)	0.31
Bleeding tendency (VAS-Score)	3,7 ± 1.8	3.2 ± 2.2	0.48
Drainages in retromuscular space			
0	0	19 (90.5)	
1	10 (47.6)	1 (4.8)	
2	11 (52.4)	1 (4.8)	< 0.001
Drainages in subcutaneous space			
0	3 (14.3)	4 (19.1)	
1	13 (61.9)	14 (66.7)	
2	5 (23.8)	3 (14.3)	0.71
Preoperative antibiotic prophylaxis			
Cefazoline	19 (90.5)	21 (100)	0.35
Cefuroxime	1 (4.8)	0	
Clindamycin	1 (4.8)	0	
Epidural anesthesia	1 (4.8)	2 (9.5)	0.55

Data are reported as absolute numbers (percentage), median (min-max) or mean (± standard deviation); p-values are calculated using the *U* test (Mann-Whitney-Wilcoxon) for continuous data, t-test for normal distribution, or Pearson´s chi-squared-test for categorical data

*VAS*, Visual analog scale (0–10)

## Results

**Fig. 1:**
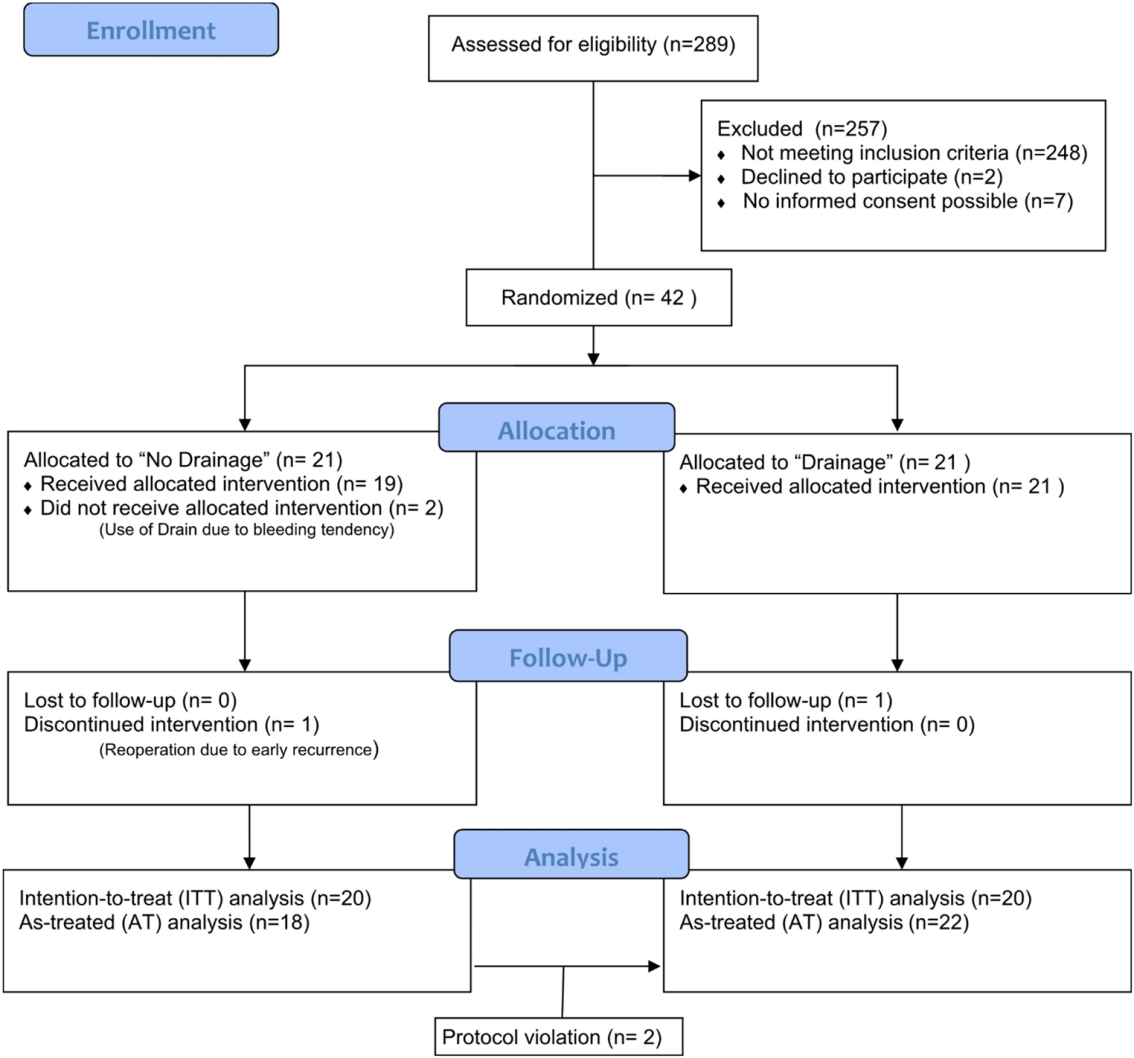
CONSORT flow chart diagram

A total of 42 patients were randomly assigned to drainage or no drainage of the rectus sheath group. Two patients were excluded from the analysis because they were lost to follow-up or required early reoperation because of a fascial burst. Therefore, the ITT population comprised 40 patients, two of whom were not treated according to the protocol and were included in the AT analysis accordingly (Fig. 1).

Patient characteristics of the study groups were well-balanced (Table 1). The mean age and body mass index of the participants were 57.8 years and 30.2 kg/m^2^, respectively. Three and five patients had a primary hernia in the group with and without drainage, respectively; the remaining patients were treated for incisional hernias.

The procedure characteristics were also well-balanced (Table 2). There were no differences regarding the size of the hernia, mesh, wounds, operative time, degree of adhesiolysis, or bleeding tendency. Meshes with a mean size of 180 cm^2^ (12 × 15 cm) were used. Subcutaneous drainage was used for 83.3% of the patients; however, this number did not differ between groups.

Ultrasonography performed on PODs 14 and 30 showed significantly lower volumes of RFC in the drainage group during ITT analyses (Table 3). The ITT analysis indicated that the median volume of RFC was reduced by 75.2% on POD 14, and by POD 30, no RFC were found. The number of patients with RFC considered clinically relevant was also significantly lower in the drainage group. This was consistent in both the ITT and AT groups.

**Table 3:** Postoperative retromuscular fluid collections (RFC)

Intention-to-treat analysis	Randomized to drainage in retromuscular space		
	Yes	No	p-value
	20 (50.0)	20 (50.0)	
Follow-up POD 14			
Volume of RFC (mL)	0.2 (0–68)	25.8 (0–259.8)	< 0.01
Patients with RFC ≥ 5 mL	4 (0.2)	12 (0.6)	0.02
Follow-up POD 30			
Volume of RFC (mL)	0 (0–65.5)	4.0 (0–283)	0.01
Patients with RFC ≥ 5 mL	1 (0.1)	8 (0.4)	0.02
**As-treated analysis**	Drainage in retromuscular space applied		
	Yes	No	p-value
	22 (55.0)	18 (45.0)	
Follow-up POD 14			
Volume of RFC (mL)	0.2 (0–68)	28.8 (0–259.8)	< 0.001
Patients with RFC ≥ 5 mL	4 (18.2)	11 (61.1)	0.01
Follow-up POD 30			
Volume of RFC (mL)	0 (0–65.5)	4.6 (0–283)	0.003
Patients with RFC ≥ 5 mL	1 (4.5)	8 (44.4)	< 0.01

Data are reported as absolute numbers (percentage) or as median (min-max); p-values are calculated using the U test (Mann-Whitney-Wilcoxon) for continuous data, Pearson´s chi-squared-test for categorical data

*POD*, postoperative day; *RFC*, retromuscular fluid collection, fluid collections dorsal of the linea alba and anterior rectus fascia

**Fig. 2:**
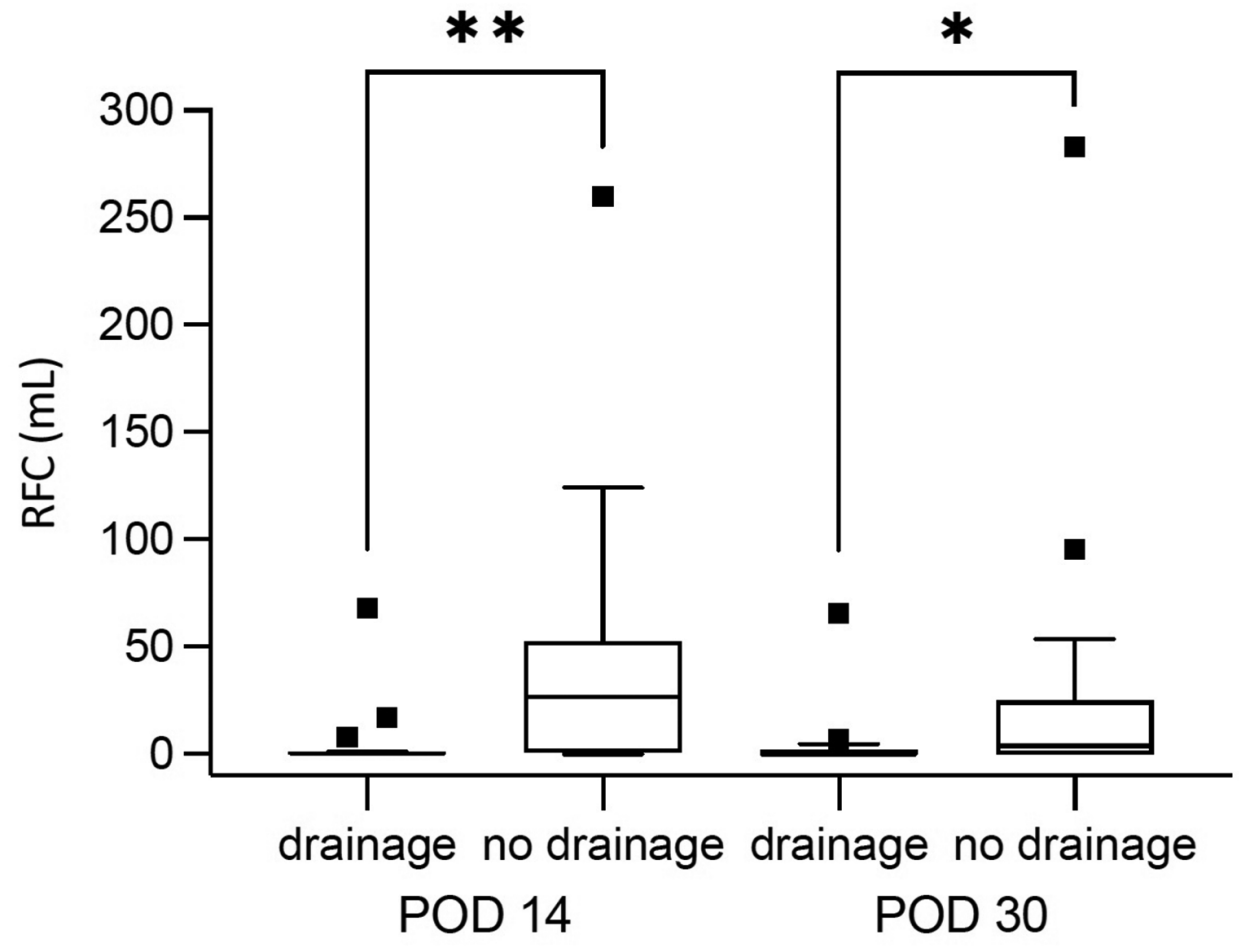
Box-plot diagram of fluid collections dorsal of the linea alba and anterior rectus fascia *POD*, postoperative day; *RFC*, retromuscular fluid collection

The use of retromuscular drainage was a significant predictive variable in logistic regression on PODs 14 (coefficient, -1.01; 95% confidence interval [CI], -1.52 to -0.50; *p* < 0.001) and 30 (coefficient, -0.62; 95% CI, -1.09 to -0.15; *p* = 0.01 (Fig. 2). The multivariable analysis used was randomized to drainage, renal disease, bleeding tendency, diabetes mellitus, adhesiolysis, American Society of Anesthesiologists score, therapeutic dosage of LMWH, and mesh size in the final model. However, being randomized to drainage was the only significant independent predictor (*p* = 0.001).

Even though the necessity for opioid analgesics and the maximum pain score of the visual analog scale did not differ between the groups on certain PODs, in the group without drainage, analgesic therapy was switched to on-demand medication earlier (Table 4). The last drain (including the subcutaneous drain) was removed significantly earlier in the group without drainage in the rectus sheath, resulting in shorter LOS.

**Table 4:** Postoperative treatment

	Randomized to drainage in retromuscular space		
	Yes	No	p-value
	20 (50.0)	20 (50.0)	
Necessity of opoid analgetics			
POD 1	5 (25.0)	6 (30.0)	0.45
POD 2	2 (10.0)	0	0.19
POD 3	1 (5.0)	0	0.36
Last day of fix analgetic therapy	6 (0–9)	3 (0–10)	0.03
Maximum Pain POD 1–3 (VAS-Score)	2.5 (0–6)	3 (0–7)	0.88
Removal of last drainage (POD)	6 (2–21)	3.5 (2–8)	0.005
Length of hospital stay (days)	6.5 (3–11)	4 (3–22)	0.01

Data are reported as absolute numbers (percentage) or as median (min-max); p-values are calculated using Pearson´s chi-squared-test for categorical data or U test (Mann-Whitney-Wilcoxon) for continuous data

*POD*, postoperative day; *VAS*, Visual analog scale (0–10)

**Table 5:** Postoperative complications

	Randomized to drainage in retromuscular space		
	Yes	No	p-value
	20 (50.0)	20 (50.0)	
Last day of follow up (POD)	49.5 (21–752)	32.5 (28–459)	0.92
Procedure related complications			
Retromuscular hematoma	0	2 (10.0)	0.49
Reoperation	0	1 (5.0)	
Needle aspiration	0	1 (5.0)	
Surgical Site infection	0	0	
Superficial, subcutaneous Seroma	11 (55.0)	9 (45.0)	0.53
Reoperation, Drainage	2 (18.2)	0	
Needle aspiration	2 (18.2)	2 (22.2)	
No therapy	7 (63.6)	7 (77.8)	
General complications			
Pneumonia	1 (5.0)	1 (5.0)	1.00
Readmission	1 (5.0)	2 (10.0)	0.55

Data are reported as absolute numbers (percentage) or as median (min-max); p-values are calculated using the U test (Mann-Whitney-Wilcoxon) for continuous data, Pearson´s chi-squared-test for categorical data

*POD*, postoperative day

Two patients in the group without retromuscular drainage had to be treated due to a large, deep hematoseroma. One underwent revision surgery on POD 34, and the other underwent partial evacuation by needle aspiration. No treatment due to RFC was necessary for patients in the group with retromuscular drainages.

Subcutaneous seromas in 20 patients (47.6%) in both groups were the most frequent finding in our study cohort. There were no significant differences between the two groups (*p* = 0.53). Two patients in the drainage group and no patient in the group without drainage required surgery. In each group, two seromas were treated with needle aspiration; the remaining patients required no treatment.

No SSIs occurred in the study population. Postoperative pneumonia occurred in one patient in each group. Pneumonia was the reason for one readmission in the group with drainage. Readmissions in the group without drainage were due to rectus sheath hematoma (*n* = 1) and unspecified abdominal pain (*n* = 1).

## Discussion

In this randomized controlled trial, we found that the use of retromuscular drains after hernioplasty with sublay mesh reinforcement reduced the number and volume of RFC on PODs 14 and 30. This was confirmed in both ITT and AT analyses. However, the group without drainage had shorter LOS and earlier reductions in pain medication requirements.

Recent prospective studies have addressed the issue of drain insertion. One randomized controlled trial included 144 patients with and without drainage and controlled for fluid collections on POD 30 using ultrasound. The group with drainage showed fewer surgical complications and wound dehiscences [28]. However, retromuscular and subcutaneous drains were not differentiated. Fluid collections were detected in 60.3% and 62% (*p* = 0.84) of patients with and without drains, respectively. This is probably due to the high frequency of subcutaneous superficial fluid collections, which is also evident in our study. The number of complications in that trial appears high for elective procedures, with up to 42.7% and 9.3% for surgical complications and wound dehiscence, respectively.

Overall, there have been few randomized studies on this topic to date, most of which do not differentiate between retromuscular and subcutaneous mesh placement [17, 18]. A non-randomized study categorized participants into groups according to the position of drainage. In the group with retromuscular drainage (*n* = 20), SSO was found significantly more frequently in 50% of the cases, and all groups with drains had a longer LOS [14]. No difference was found concerning SSI. This is partially contrary to our results, as we found no SSI in the entire cohort and detected SSO affecting the retromuscular layer only in the group without drainage.

A retrospective trial evaluated 581 patients with retromuscular mesh reinforcement and found no difference regarding SSIs when drains were used; however, fewer noninfectious surgical site complications were observed [13]. Furthermore, a retrospective trial addressing the drain duration for 117 patients with 64 SSIs showed no relationship between the POD when the last drain was removed and seroma development, but the SSI incidence increased [29].

No incidence of SSI occurred in our study population, probably because only midline hernias without lateral release were included in our study. We recently found that the incidence of SSI was higher for the entire population after sublay hernioplasty at our hospital [30]. However, in the current trial, we did not observe a difference in the incidence of SSIs, although the trial was not powered for this endpoint. In the group without drainage, one patient required subsequent reoperation, and one required needle aspiration because of a large retromuscular hematoseroma. This could possibly have been avoided by retromuscular drainage. However, there is no statistical correlation. As SSO and revision surgery were not a primary endpoint of this study, the number of cases is too small to draw any conclusions. On the other hand, 83.3% of the patients in this study had one or more drains inserted into the subcutaneous site. Nevertheless, a subcutaneous seroma occurred in 47.6%, which required treatment in 14.3%. Therefore, a considerable proportion of treatment failures can also be assumed for the retroprosthetic site. Overall, a large registry study demonstrated more complication-related reoperations after drain insertion [5].

Shorter LOS was one benefit observed for the group without drainage in our study, which was also shown in other studies [13, 14]. However, this prolonged LOS is presumably caused by the inserted drains. This could be remedied by outpatient care with the drain in place. In addition, a recent retrospective study demonstrated that a retromuscular drainage removal, irrespective of the flow rate, shortens the LOS without increasing the complication rate [31].

A limitation of the current study is that the ultrasonography-based volume measurements have the risk of underestimation [32]. However, this method was also used in other randomized studies to quantify collections and appears to be reliable [28]. Moreover, all measurements were performed in the same manner by one trained person who was blinded to patient allocation. All measurements were performed before closing the database and calculating the study endpoints, and then documented. Therefore, the risk of bias in this respect was reduced. In addition, blinding may be limited as drainage tubes leave scars. However, since subcutaneous drains were inserted in nearly all cases and no distinction can be made between scars of subcutaneous and retromuscular drains, it seems possible to assume that the investigator was largely blinded. A third limitation is the use of a self-adhesive mesh. This could influence the formation of RFC, so that the generalizability may be limited. This study also included primary and incisional ventral hernias, which may lead to inhomogeneity. However, only patients with incisional hernias after midline laparotomy without previous hernia surgery and without dissection or concomitant defects of the rectus sheath were included. This seems to avoid a significant influence. This was also the case in the most recently published studies on this topic [29, 15]. In addition, the width of a pre-existing rectus diastasis was not recorded pre- and intraoperatively. This influences the required mesh size and overlap and can therefore have an impact on the formation of RFC.

The sonographic detection of fluid collections leads to the question of clinical relevance. In our study, RFC was detected more frequently in the group without drainage. However, the majority developed no relevant seromas and the need for analgesics was lower and the LOS shorter. The measured volume of RFC decreased spontaneously after 30 days in almost all cases. There is no statistically significant correlation for SSO, although the number of cases in this study is insufficient for this endpoint. While this study addresses the local periprosthetic changes, a study powered for an endpoint such as SSO would be of higher clinical relevance. However, this leads to a sample size of at least 480 patients requiring a large multicenter study with extensive funding.

Therefore, our data suggest that the insertion of retromuscular drains reduces the number and size of retromuscular, periprosthetic fluid collections. Whether hematoseroma requiring treatment could have been avoided by inserting drainage cannot be concluded from our dataHowever, it should be noted that the majority of patients without retromuscular drainage did not develop complications despite detectable RFC. Therefore, it seems advantageous to identify risk factors for developing complications after RFC in order to identify a low-risk group that benefits from a shorter LOS by not using drains. Given the current weak evidence and controversial study results, further randomized studies are required, to further evaluate the clinical relevance of these findings.

## Conclusion

We found that the use of retromuscular drainage for sublay hernia repair reduced periprosthetic fluid collections in our population but prolonged hospital stay. Whether the use of drains and the reduction of RFC can prevent SSO or revision surgery cannot be determined from our data, the relevance is therefore not assessable. However, not using drains did not increase the risk of infection in our population. Therefore, further larger studies are required to determine the clinical relevance of drains.

## Data Availability

No datasets were generated or analysed during the current study.
